# Assessment of 3D printed mechanical metamaterials for prosthetic liners

**DOI:** 10.1177/09544119231225529

**Published:** 2024-01-27

**Authors:** Kirstie M Devin, Jinghua Tang, Andrew R Hamilton, David Moser, Liudi Jiang

**Affiliations:** School of Engineering, Faculty of Engineering and Physical Sciences, University of Southampton, Southampton, UK

**Keywords:** Lower limb amputee, prosthetic liner, metamaterials, pressure distribution, 3D printing, finite element analysis

## Abstract

This study focuses on novel design and evaluation of Elastic 50A (EL50) mechanical metamaterials with open-cell patterns for its potential application to lower limb residuum/socket interfaces, specifically that of a transtibial (TT) amputee. Mechanical characteristics, that is, effective Young’s modulus (*E*), was tuned by altering metamaterial porosity, which was experimentally verified. Specifically, pore radius of the unit cell was varied to achieve a range of *E*-values (0.05–1.71 MPa) for these 3D printed metamaterials. Finite Element Analysis (FEA) was conducted to evaluate pressure distribution across key load-bearing anatomical sites of a TT residuum. Using designed metamaterials for homogeneous liners, pressure profiles were studied and compared with a silicone liner case. Additionally, a custom metamaterial liner was designed by assigning appropriate metamaterials to four load-sensitive and tolerant anatomical sites of the TT residuum. The results suggest that lowest pressure variation (PV), as a measure of pressure distribution levels and potential comfort for amputees, was achieved by the custom metamaterial liner compared to any of the homogeneous liners included in this study. It is envisaged that this work may aid future design and development of custom liners using now commonly available 3D printing technologies and available elastomer materials to maximise comfort, tissue safety and overall rehabilitation outcomes for lower limb amputees.

## Graphical abstract

**Graphical abstract:**
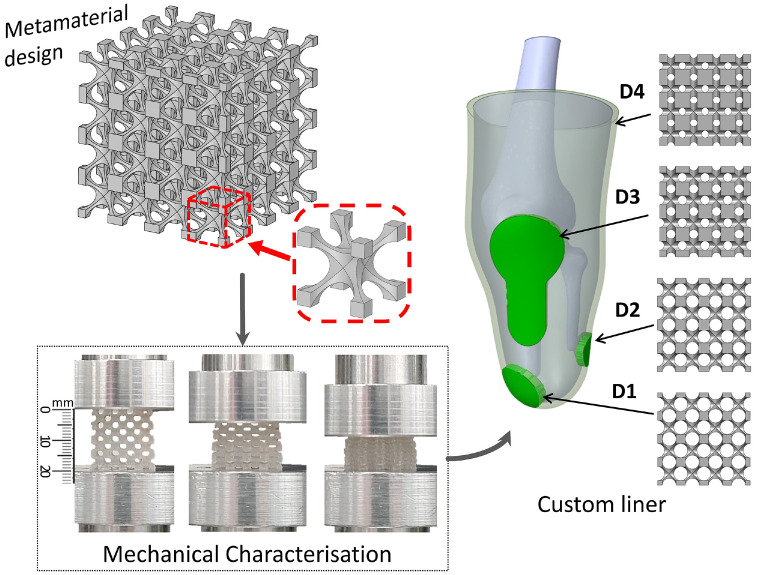

## Introduction

The interface between the lower limb residuum and the prosthetic socket is critical to facilitate effective load transfer and ensure user stability and comfort during activities of daily living.^1^ Residuum tissues are not biologically accustomed to endure prolonged exposure to multidirectional forces. The presence of scar tissue can further compromise its tolerance to external loading. Prosthetic liners, typically made of silicone or polyethylene foam, are often worn by amputees to provide cushioning and protection to residua tissue from the hard socket wall.^1^

Mechanically compliant liners help distribute load across the residuum/socket interface. Even pressure distribution is known to be desirable for socket comfort and thus overall rehabilitation outcomes.^2^ Even pressure distribution is also the underlying principle for hydrostatic sockets^2,3^ which have been widely used for transtibial (TT) amputees. However, achieving relatively even load distribution through liner designs has proved challenging, particularly for TT amputees due to the existence of bony prominences (e.g., tibial end, fibula head and tibial tuberosities).^4^ Consequently, interface stresses can vary significantly across different TT residuum anatomical sites.^5^ For instance, Sanders et al.^4^ reported interface pressure of up to 224 kPa at anterior-distal sites (contains a bony prominence) compared to 115 kPa at the popliteal fossa (PF) site (no bony prominence). Laing et al.^6^ also reported pressure of up to 173 kPa at anterior-distal and 86 kPa at PF sites. The uneven distribution of these stresses, especially elevated loading at load-sensitive bony prominence sites, is known to affect comfort and may increase the risk of tissue injury (e.g., ulceration).^3,7,8^

Although prosthetic sockets are usually bespokely made to accommodate user-specific characteristics (e.g., residuum shape, tissue mechanical properties, painful sites),^9^ most conventional prosthetic liners are batch-produced. In order to help distribute interface load, thus improve comfort and overall rehabilitation outcomes, custom liners have been reported,^9^ though most of them focus on adapting to the shape of the residuum while still using conventional silicone materials.^10^ Many studies have been conducted to understand the effect of liner material and geometry on interface load distribution with a view to improve load distribution.^11,12^ A recent review^9^ suggests that changing localised liner material stiffness has proved effective in helping load redistribution. However, current manual approaches to achieve this, for example, cutting out ready-made silicone liners at load-sensitive sites then replacing them with silicone of different thickness,^9,10^ is an inefficient means of producing patient-specific liners. The sole use of conventional silicone materials also limits material choices, design freedom and manufacturability to achieve an even load distribution for personalised liners.

3D printing has been exploited as a promising means of producing prosthetic sockets^13^ and moulds^14^ for custom silicone liners, as it can accommodate complex residuum shapes in design and manufacturing to an appropriate level of accuracy.^15^ Advancements in additive manufacturing technology means that a more comprehensive range of elastomeric materials exhibiting a wider range of stiffness, as compared with that of silicone, can now be exploited. On the other hand, the inception of elastomeric mechanical metamaterials has attracted significant interest in recent years, as their mechanical properties (e.g., geometry and stiffness) can be tuned by altering the architecture of unit cells and structures while still maintaining usage of the same base material.^15,16^ 3D printed elastomers^17^ in particular show great promise for a range of biomedical applications due to high flexibility, strength and variable stiffness for applications involving complex surface contours such as TT residua in this study. Indeed, Brown et al.^18^ reported an initial study involving the use of 3D printed TangoPlus^19^ metamaterials as interface materials to offload pressure at the residuum/socket interface. However, there lacks systematic studies on the design and evaluation of prosthetic liners, especially custom liners.

This paper reports a preliminary study focusing on design and development of mechanical metamaterials based on Elastic 50A® (EL50)^20^ elastomeric material, which is widely accessible for table-top 3D printers. The specific aim is to improve the distribution of pressure across anatomical sites of a TT residuum. Metamaterials made of elastomeric material were designed based on an open unit cell with varying porosity in order to achieve different stiffnesses. The mechanical properties of the metamaterials were experimentally evaluated. Finite Element Analysis (FEA) was used to assess pressure distribution across the TT socket interface for homogeneous liners. A novel custom liner was subsequently designed to comprise different metamaterials across residuum anatomical sites with a view to improve pressure distribution. It is envisaged that this may help shed light on the means and potential of exploiting table-top 3D printers and associated elastomeric metamaterials for future development of personalised prosthetic liners.

## Materials and methods

### Design and fabrication of metamaterials

**Figure 1.**
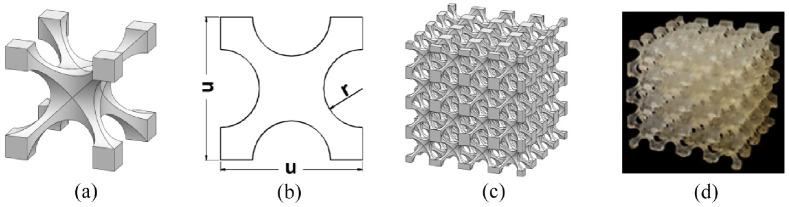
Schematics showing (a) a design of a typical 3D cellular unit, (b) cross-sectional view of the 3D cellular unit and (c) a metamaterial formed by stacking 3D cellular units. (d) A printed metamaterial.

A simplified 3D cellular unit was chosen for the metamaterial design, as shown in Figure 1(a). Such cellular unit is well-known for achieving a wide range of effective Young’s modulus (*E*) (1–1000 kPa)^21^ and therefore have been used for body support applications.^7,22,23^ The 3D cellular unit is defined by two critical design parameters, that is, the cell size (u) and pore radius (r), as illustrated by the cross-sectional view (Figure 1(b)). Both u and r affect the relative density (*ρ_r_*) and porosity (φ) of the 3D cellular unit,^24^ thus affecting E. In this study, a typical cell size u = 5 mm was chosen. This falls in the range of typical liner thickness of approximately 3–10 mm^11,25^ while liners of progressive thickness can also reach up to 16 mm distally.^26^ Furthermore, it is not uncommon to use cellular materials consisting of a singular or few cell layers.^27^ The 3D cellular units were stacked in all directions to produce isotropic metamaterial samples with a dimension of 20 × 20 × 20 mm (Figure 1(c) and (d)). The sample dimensions were chosen based on the consideration of BSI Standards for compression tests of rubber-like materials,^28^ whereby an aspect ratio of greater than or equal to one is recommended. Such sample dimensions are also sufficiently larger than the unit cell size (u = 5 mm), which was recommended^27^ for experimental evaluation of E for open-cell foams.

The theoretical porosity (φ_T_) of the metamaterials was evaluated based on equation (1), using SolidWorks 2022 (Dassault Systèmes, France), where V_p_ represents the volume of pores and V_s_ is the solid volume.

(1) $$\varphi_{T}=\frac{V_{p}}{V_{p}+V_{s}}\times 100\%$$

Table 1 shows that φ_T_ changes with the variation of r, while u was held constant at 5 mm. The range of r was selected in such way in order to obtain the appropriate range of metamaterial porosity in this study.

**Table 1.** Design and theoretical porosity of metamaterials.

Design	*u* (mm)	*r* (mm)	φ_*T*_ (%)
D1	5	1.50	83
D2	5	1.37	76
D3	5	1.00	50
D4	5	0.80	35

**Figure 2.**
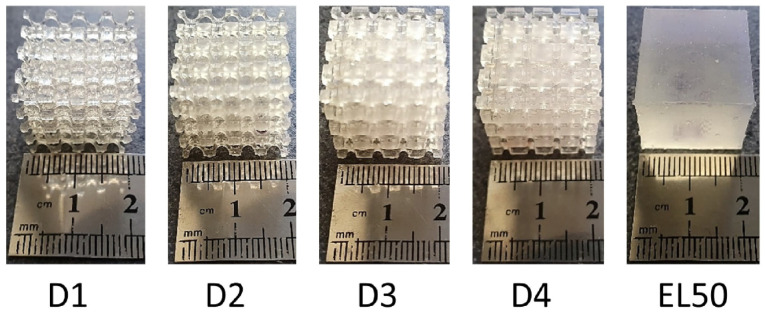
Fabricated samples.

The designed metamaterials along with solid EL50 samples were 3D printed using a stereolithography Form 3 printer (Formlabs Inc., Germany), as shown in Figure 2. The 3D printed samples were then washed using isopropyl alcohol for 20 min and subsequently cured for 20 min at 60°C, as recommended by the manufacturer.

### Characterisation of metamaterials

Densities of metamaterials (*ρ_m_*) and solid EL50 (*ρ_EL50_*) were obtained from measured volume and mass of the samples. Relative density (*ρ_r_*) is subsequently defined in equation (2),^29^ which is known for cellular structures. φ_e_ represents experimentally induced porosity.

(2) $$\rho_{r}=\frac{\rho_{m}}{\rho_{\mathrm{EL}50}}=1-\varphi_{e}$$

Table 2 shows measured *ρ_m_* and corresponding *ρ_r_* and φ_e_ which were calculated based on equation (2). The theoretical porosity, that is, φ_T_ (equation (1)) is also included and show good alignment with φ_e_. The minor discrepancies may be caused by slight variations in manufacturing (e.g., pore radius slightly differing from theoretical designs).

**Table 2.** Comparison of designed metamaterials.

Design	D1	D2	D3	D4	EL50
ρ_*m*_ (kgm^−3^)	172	277	585	737	1018
ρ_*r*_	0.17	0.27	0.58	0.72	1.0
φ_*e*_ (%)	83	73	42	28	0
φ_*T*_ (%)	83	76	50	35	0

**Figure 3.**
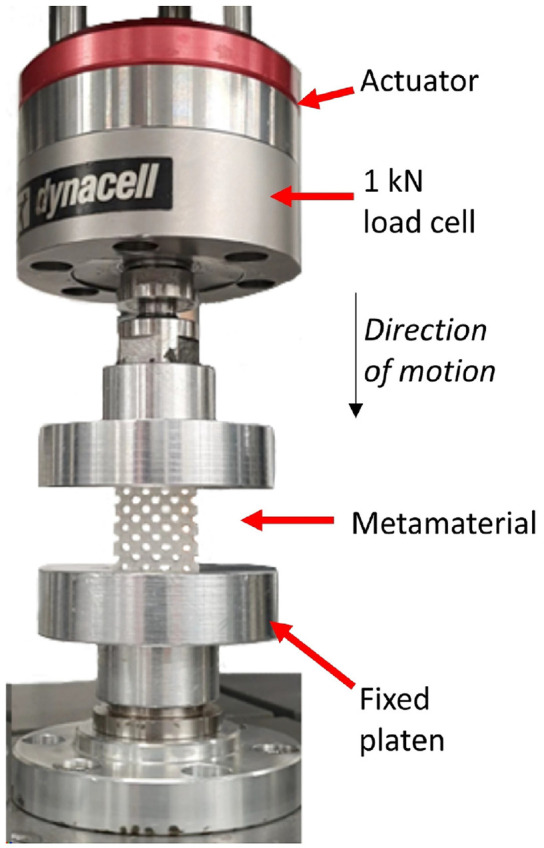
A typical compressive loading test setup.

Mechanical compressive loading tests were performed on the samples using a uniaxial material test machine (ElectroPuls E1000, Instron, Illinois Tool Works Inc., US) and a typical test setup is shown in Figure 3. Compressive load of up to 80 N was applied to each sample while corresponding strain was measured simultaneously. This is equivalent to a peak pressure of 200 kPa, which has been commonly reported^4^ at TT residuum/socket interfaces. At least four repeated tests were conducted for each sample and the mean of stress-strain curves were produced. *E*-values were obtained through linear fittings of the stress-strain curves.

### FEA of the TT residuum/socket interface

**Figure 4.**
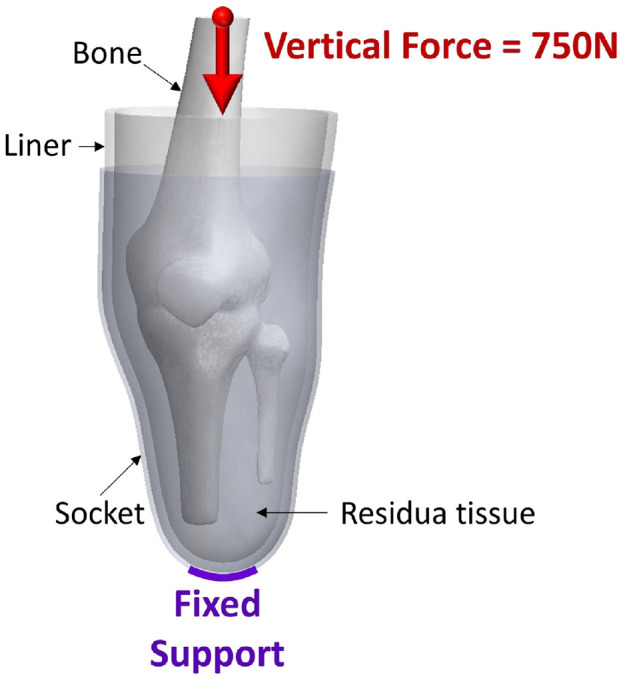
Boundary conditions applied to interface components.

FEA was conducted using ANSYS 2021R2 (ANSYS Inc., US) to assess pressure distribution across a residuum/socket interface when liner materials with varied *E* were assigned. Models were constructed using MeshMixer 3.5 (Autodesk Inc., US) and SolidWorks. A 3D TT residuum model was established by scanning a positive cast of a TT residuum. A truncated TT bone module model was created in accordance with surgical guidelines of TT amputations and a detailed description was reported previously by McGrath et al.^30^ Subsequently, a bone cavity was created within the residuum to host the truncated bone model. The solid liner was constructed by extruding the exterior surface of the residuum^18^ uniformly by 5 mm in the direction normal to the residuum. This thickness is equivalent to a single layer of the unit cell (u = 5 mm) as shown in Figure 1(a). The socket was constructed likewise, except the exterior liner surface was used as the extrusion reference. This ensured liner/residuum and liner/socket interfaces were flush against each other to emulate in-vivo socket interface interactions.^18^ The complete 3D model for FEA is shown in Figure 4.

A fixed support was applied to the socket’s distal-end while a vertical load of 750 N was exerted on the bone. This equates to a peak vertical ground reaction force during ambulation from an amputee with an average bodyweight of approximately 70 kg. It was reported^31^ that the peak vertical ground reaction force is approximately 110% of that of bodyweight. Bone/residuum and residuum/liner interfaces were assumed bonded and a coefficient of friction of 0.5 was applied to the liner/socket interface to represent a sticky surface between liner and residuum and greater sliding between liner and socket, which are commonly used to simulate elastomeric liners.^31^ Table 3 lists material properties,^31–33^ that is, *E* and Poisson’s ratio (ν), used in FEA including those for a typical silicone liner.^11^

**Table 3.** Material properties used in FEA.

Component	*E* (MPa)	ν
Bone	15,000	0.30
Residua tissues	0.20	0.45
Silicone	0.28	0.49
Socket	1,290	0.30
EL50	2.63	0.49
Metamaterials	0.05–1.71	0.49

The range of *E*-values of metamaterials were assigned to the solid liner layer of 5 mm thickness based on experimental data obtained from compressive loading tests on 20 × 20 × 20 mm samples (Figure 2). It is important to note that the stiffness of a single layer of unit cells may vary slightly compared with respective bulk test samples based on a study of the mechanical behaviour of foams with random (Voronoi) pore size and morphology.^27^ However, while the distribution of pore size and morphology play a key role in size effects on stiffnesses for Voronoi microstructures, for the unit cell designs adopted in this work (as shown in Table 2), the distribution of pore size and morphology are nominally the same in both single cell and test sample cases. It is thus plausible to assume that there is little size effect on stiffness in this work. Therefore, *E* obtained from corresponding compression experiments were used as liner stiffnesses in FEA. This also ensures the primary focus to be on the relative comparison across different metamaterial designs, that is, D1–D4. ν of 0.49 was used for metamaterials in this study since isotropic open-cell structures were reported to exhibit ν of close to 0.5 at low densities.^29,34^ Therefore, ν of 0.49 were assigned for all liner materials in this study as they are all highly elastic materials.

FEA was conducted considering homogenous liner cases first, whereby corresponding *E*-values of metamaterials, silicone and EL50 were assigned to the entire liner region, respectively. Pressure distribution across anatomical sites of the TT residuum were studied. Based on these comprehensive studies, a novel custom liner was subsequently designed comprising metamaterials with differing *E*-values at four different anatomical sites of the TT residuum. Specifically, different metamaterials were assigned to four identified liner regions corresponding to the distal tibial-end (DTE), distal fibula-end (DFE), patella tendon (PT) and popliteal fossa (PF) sites, which are known load bearing and sensitive sites for TT residua.^32,35^ The shapes and geometry of the respective liner regions were chosen based on commercially-available pressure pads^36^ that are commonly used in clinics to help improve socket comfort for TT amputees.^2,36,37^ Pad locations were centred around the pressure concentration point, that is, the highest pressure location in each specific region. Within each liner region, the *E*-value was kept the same as that of the assigned metamaterial.

Pressure variation (PV) was introduced as a measure of pressure distribution whereby lower PV corresponds to a more evenly distributed profile, as shown in equation (3), where $x_{i}$ represents peak pressure obtained at each site and $\bar{x}$ represents the mean of peak pressure across selected sites.

(3) $$\mathrm{PV}=\sqrt{\frac{\sum {\left(x_{i}-\bar{x}\right)}^{2}}{n}}$$

In this work, peak pressure at four anatomical sites, that is, DTE, DFE, PT and PF, were extracted from FEA results in order to obtain PV, thus *n* = 4 was used. Peak pressure was obtained at the same sites for all FEA studies, ensuring validity of PV comparisons.

## Results and discussion

### Mechanical characterisation of metamaterials

**Figure 5.**
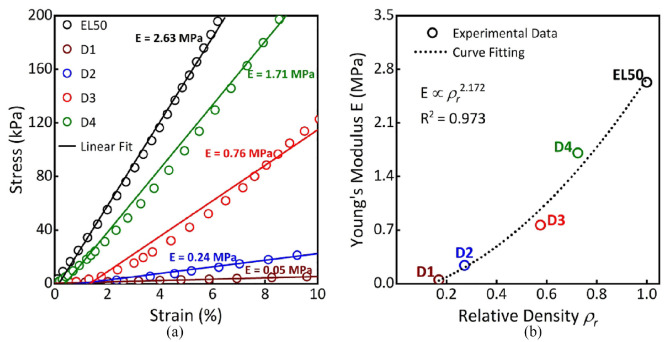
(a) Compressive stress-strain curves. (b) *E* of different metamaterial designs as a function of ρ_*r*_ with power-law fitting.

Figure 5(a) shows stress-strain curves obtained from compressive loading tests including the corresponding linear fittings. Figure 5(b) shows that *E* increases as *ρ_r_* increases. Curve fitting indicates a power-law relationship between *E* and *ρ_r_* (*R*^2^ > 0.973), as shown in equation (4), where *n* is a constant dependent on the structure.^24,29^

(4) $$E\;\propto \;{\rho_{r}}^{n}$$

Exponent (*n*) of close to 2 (*n* = 2.17) in Figure 5(b) aligns well with those typically observed for open-cell foams,^34^ exhibiting bending-dominated deformation mode for these metamaterials. This indicates a significant decrease in *E* as *ρ_r_* decreases.^24,29^ This experimentally validates our metamaterial design and the corresponding *E*-values that were subsequently used as inputs for FEA in this study. It is also important to note metamaterials were designed such that their *E*-values are in the approximate range of 0.05–1.71 MPa (Figure 5(a)), which roughly covers the wide range of reported tissue properties (0.05-2 MPa).^31,38^

### Pressure distribution from FEA

**Figure 6.**
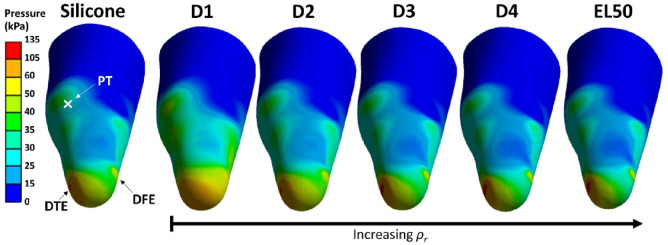
Pressure distribution with homogeneous liners made of silicone, D1, D2, D3, D4 and EL50.

Figure 6 shows pressure distribution across the residuum when using homogeneous liners made of different materials, obtained via FEA. For all cases, pressure values were relatively higher at DTE and DFE as compared with those at PT and PF. This is primarily due to the existence of the tibial tuberosity at DTE and truncated fibula bone at DFE, whereby bony prominences are responsible for elevated pressure.^4^ In particular, peak pressure at DTE (100–135 kPa) and DFE (55–61 kPa) range across different homogenous liners, which fall in the range of typical values reported at TT residuum/socket interfaces (up to approximately 200 kPa).^4,6,8^ High DTE pressure was previously linked to pain and increased risk of mechanically-induced tissue breakdown^39^ as it is clinically poor at sustaining load.^31^ Likewise, the DFE also exhibits a stress “hotspot” due to load transmission down through the fibula shaft towards the truncated fibula, resulting in accumulative pressure. Although comparatively lower than that at DTE, it is equally important to prevent stress accumulating at this site as it too cannot tolerate high loading.^32^ In contrast, peak pressure ranges were comparatively lower at PT (37–43 kPa) and PF (30–35 kPa) sites when different homogenous liners were adopted in FEA. PT and PF are known key load-bearing sites whereby pressure of up to approximately 100 kPa^8^ and 115 kPa^4^ have been reported, respectively. The PF is also known for its ability to redistribute pressure due to the existence of the gastrocnemius muscle (i.e., large tissue presence),^37^ which explains why obtained pressure was the lowest of the four sites.

**Figure 7.**
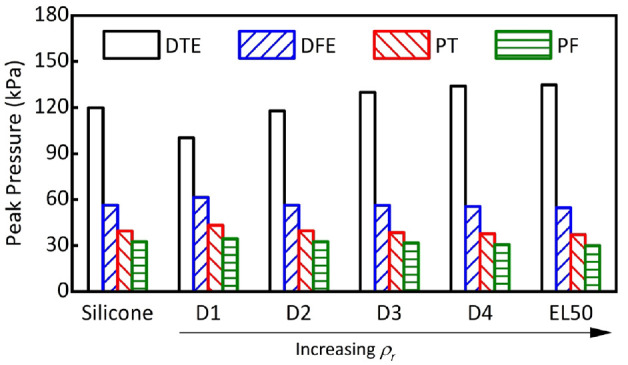
Peak pressure obtained at the DTE, DFE, PT and PF of the residuum as a function of liner materials.

Figure 7 compares peak pressures obtained from FEA using different liner materials. D1 resulted in lowest peak pressure at DTE (100 kPa) but highest at the DFE site (61 kPa). Lower pressure at DTE is expected for D1 liner due to its low stiffness (*E* = 0.05 MPa), that is, the most compliant material as compared with all other counterparts. The pressure increase observed at DFE, PT and PF further indicates the shift of loading profiles and thus demonstrates the importance of considering pressure distribution across residuum loading sites as a whole.

**Figure 8.**
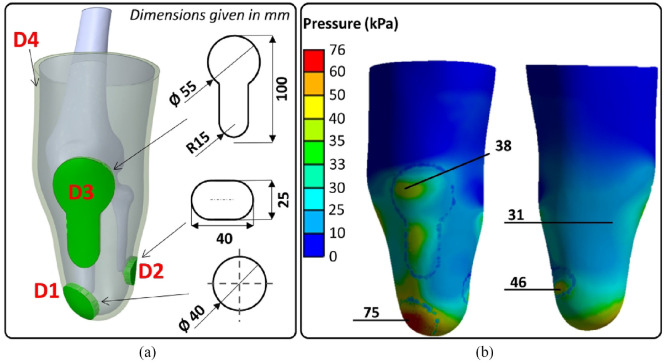
(a) A custom liner with metamaterials applied at the four sites. (b) Pressure distribution across the residuum.

A novel custom liner was thus designed in order to achieve pressure distribution, that is, lowest PV across DTE, DFE, PT and PF sites. Different metamaterials were assigned to four identified liner regions as shown in Figure 8(a). Previous findings suggest^9,11^ that an ideal liner should possess higher *E* at sites with greater tissue presence to limit relative motion, and lower *E* at sites of high stress concentration to alleviate pressure. We thus assigned D1, D2, D3 and D4 to DTE, DFE, PT and PF sites, respectively. In essence, metamaterials with lower *E* were assigned to anatomical sites associated with relatively higher pressure, with a view of achieving lower PV, that is, improved pressure distribution. In particular, D1 (lowest *E* of 0.05 MPa) was assigned to DTE (highest pressure concentration site), while D4 (highest *E* of 1.71 MPa) was assigned to the remaining residuum region, as shown in Figure 8(a), which encompasses the PF (lowest pressure site). Figure 8(b) shows the pressure distribution resulting from FEA for the custom-designed metamaterial liner.

**Figure 9.**
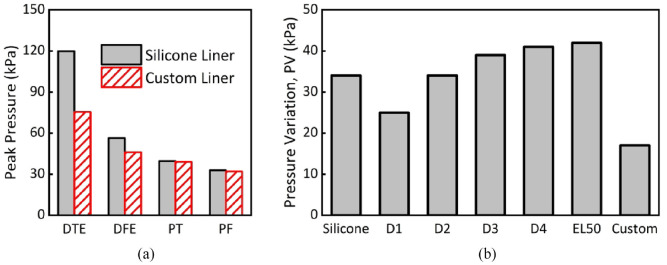
(a) Peak pressure obtained at the four sites for the silicone liner versus the custom liner and (b) PV for the homogeneous liners compared to the custom liner.

Figure 8(b) shows that peak pressures at DTE (75 kPa), DFE (46 kPa), PT (38 kPa) and PF (up to 31 kPa) were obtained for the custom liner case. In comparison to homogeneous liner cases (Figure 6), pressure decreased substantially at DTE and DFE bony prominence sites, while pressure remained similar at PT and PF load-bearing sites. Figure 9(a) specifically compares FEA peak pressure values between the custom metamaterial liner and its typical silicone counterparts. The custom liner resulted in a reduction in pressure at DTE (75 kPa vs 120 kPa) and DFE (46 kPa vs 56 kPa), with no notable change at PT and PF. Figure 9(b) shows PV for the custom liner compared to that of silicone and other homogenous liner cases. It is important to note that the custom liner revealed the lowest PV (approximately 17 kPa) compared to any other homogeneous liner (Figure 9(b)). In particular, PV is even lower than that of the “softest” liner (D1, *E* = 0.05 MPa), further demonstrating the need for customised liners. Thus, a custom metamaterial liner could be designed and printed to achieve a more even pressure distribution across the residuum in order to improve socket comfort and tissue safety.^3^ This allows effective pressure offloading from load-sensitive (e.g., DTE and DFE) to load-tolerant sites (e.g., PF).

Based on this preliminary research, optimisation of metamaterial designs and properties could be conducted to further reduce PV and potentially accommodate a wider range of bespoke residua profiles and tissue properties. This could be potentially achieved at the personalised liner design stage, whereby metamaterials with a wide range of stiffnesses could be achieved by further varying key unit cell design parameters such as pore radius and unit cell size. As such, metamaterials of different stiffness could be allocated at different anatomical regions based on bespoke loading profiles and tissue properties of each individual residuum to form a custom liner. It is envisaged that the entire liner could then be 3D printed to comprise purposely-designed liner sections with varied stiffnesses to minimise overall PV. Moreover, while only vertical load was applied in FEA in this preliminary research, in future work, it would be beneficial to apply multidirectional forces mimicking different loading phases of a gait cycle. This would allow comprehensive assessment of pressure distribution during walking. Further reduction of PV could also be conducted explored by using other 3D printing elastomers or choosing different unit cell designs. Future work is also required to conduct real-world amputee tests, whereby real-time pressure measurements at residuum/socket interfaces and level of comfort can be studied and compared when using custom liners and other homogenous liners, such as habitual silicone liners.

## Conclusions

This preliminary study involves design and evaluation of 3D printed mechanical metamaterials based on EL50 elastomer, and their potential applications as liner materials for TT residuum/socket interfaces. Open-cell unit metamaterials were designed with varying porosity to achieve a range of stiffness, that is, *E* in the range of 0.05–1.71 MPa, which were verified by experimental compressive loading tests. A power-law relationship between *ρ_r_* and *E* was identified that aligns well with the predicted relationship for open-cell structures. In the case of homogenous liners, FEA results show pressure was comparably higher at DTE (up to 135 kPa) and DFE (up to 61 kPa) sites than that at the PT (up to 43 kPa) and PF (up to 35 kPa) in all cases. Use of the most compliant material, that is, D1 (*E* = 0.05 MPa) led to notable pressure reduction at DTE. Furthermore, a novel custom metamaterial liner was designed with selective designation of metamaterials to different anatomical sites. FEA results show that lowest PV can be achieved by the custom liner as compared to use of homogeneous silicone and metamaterial liners. This potentially offers a promising means to design and develop a range of 3D printed custom liners with improved comfort and tissue safety for individual amputees.

## References

[1] Hafner BJ Cagle JC Allyn KJ , et al. Elastomeric liners for people with transtibial amputation: survey of prosthetists’ clinical practices. Prosthet Orthot Int 2017; 41: 149–156. 27613589 10.1177/0309364616661256 PMC5344787

[2] Kahle J . Conventional and hydrostatic transtibial interface comparison. J Prosthet Orthot 1999; 11: 85–91.

[3] Moo EK Osman NA Pingguan-Murphy B , et al. Interface pressure profile analysis for patellar tendon-bearing socket and hydrostatic socket. Acta Bioeng Biomech 2009; 11: 37–43. 20405814

[4] Sanders JE Lam D Dralle AJ , et al. Interface pressures and shear stresses at thirteen socket sites on two persons with transtibial amputation. J Rehabil Res Dev 1997; 34: 19–43. 9021623

[5] Petron A Duval JF Herr H . Multi-indenter device for in vivo biomechanical tissue measurement. IEEE Trans Neural Syst Rehabil Eng 2017; 25: 426–435. 27244744 10.1109/TNSRE.2016.2572168

[6] Laing S Lythgo N Lavranos J , et al. An investigation of pressure profiles and wearer comfort during walking with a transtibial hydrocast socket. Am J Phys Med Rehabil 2019; 98: 199–206. 30222605 10.1097/PHM.0000000000001043

[7] Klute GK Glaister BC Berge JS . Prosthetic liners for lower limb amputees: a review of the literature. Prosthet Orthot Int 2010; 34: 146–153. 20384553 10.3109/03093641003645528

[8] Beil TL Street GM Covey SJ . Interface pressures during ambulation using suction and vacuum-assisted prosthetic sockets. J Rehabil Res Dev 2002; 39: 693–700. 17943671

[9] Yang X Zhao R Solav D , et al. Material, design, and fabrication of custom prosthetic liners for lower-extremity amputees: a review. Med Nov Technol Devices 2023; 17: 100197.

[10] WillowWood. Custom liners, https://willowwood.com/products-services/liners/custom/ (2023, accessed 9 August 2023).

[11] Cagle JC Hafner BJ Sanders JE . Characterization of prosthetic liner products for people with transtibial amputation. J Prosthet Orthot 2018; 30: 187–199. 30906148 10.1097/JPO.0000000000000205 PMC6425736

[12] Sanders JE Nicholson BS Zachariah SG , et al. Testing of elastomeric liners used in limb prosthetics: classification of 15 products by mechanical performance. J Rehabil Res Dev 2004; 41(2): 175–186. 15558371 10.1682/jrrd.2004.02.0175

[13] Kim S Yalla S Shetty S , et al. 3D printed transtibial prosthetic sockets: a systematic review. PLoS One 2022; 17: e0275161. 36215238 10.1371/journal.pone.0275161 PMC9550041

[14] Oldfrey B Tchorzewska A Jackson R , et al. Additive manufacturing techniques for smart prosthetic liners. Med Eng Phys 2021; 87: 45–55. 33461673 10.1016/j.medengphy.2020.11.006

[15] Wang K Wu C Qian Z , et al. Dual-material 3D printed metamaterials with tunable mechanical properties for patient-specific tissue-mimicking phantoms. Addit Manuf 2016; 12: 31–37.

[16] Petrochenko PE Torgersen J Gruber P , et al. Laser 3D printing with sub-microscale resolution of porous elastomeric scaffolds for supporting human bone stem cells. Adv Healthc Mater 2015; 4: 739–747. 25522214 10.1002/adhm.201400442

[17] Bachtiar EO Erol O Millrod M , et al. 3D printing and characterization of a soft and biostable elastomer with high flexibility and strength for biomedical applications. Mech Behav Biomed Mater 2020; 104: 103649. 10.1016/j.jmbbm.2020.103649 PMC7078069 32174407

[18] Brown N Owen MK Garland A , et al. Design of a single layer metamaterial for pressure offloading of transtibial amputees. J Biomech Eng 2021; 143(5): 051001. 33493283 10.1115/1.4049887 PMC10782866

[19] Stratasys Ltd. Digital materials data sheet [online]. Minnesota, Stratasys, 2018.

[20] Formlabs Inc. Elastic 50A material data sheet [online]. Berlin, Germany: Formlabs, 2020.

[21] Jiao P Alavi AH . Artificial intelligence-enabled smart mechanical metamaterials: advent and future trends. Int Mater Rev 2021; 66: 365–393.

[22] Kang S Lee J Lee S , et al. Highly sensitive pressure sensor based on bioinspired porous structure for real-time tactile sensing. Adv Electron Mater 2016; 2: 1600356.

[23] Mohanraj H Filho Ribeiro SLM Panzera TH , et al. Hybrid auxetic foam and perforated plate composites for human body support. Phys Status Solidi B 2016; 253: 1378–1386.

[24] Hamilton AR Thomsen OT Madaleno LAO , et al. Evaluation of the anisotropic mechanical properties of reinforced polyurethane foams. Compos Sci Technol 2013; 87: 210–217.

[25] Boutwell E Stine R Hansen A , et al. Effect of prosthetic gel liner thickness on gait biomechanics and pressure distribution within the transtibial socket. J Rehabil Res Dev 2012; 49: 227–240. 22773525 10.1682/jrrd.2010.06.0121

[26] Össur. Össur prosthetics catalogue. In: Össur (ed.) Prosthetic solutions 2023 catalogue. Reykjavik, Iceland: Össur, 2023, pp. 24–45. https://www.ossur.com/en-gb (accessed 15 June 2023).

[27] Tekoğlu C Gibson LJ Pardoen T , et al. Size effects in foams: experiments and modeling. Prog Mater Sci 2011; 56: 109–138.

[28] The British Standards Institution. ISO 7743:2017. Rubber, vulcanized or thermoplastic – determination of compression stress-strain properties [online]. 5th ed. London, UK: BSI Standards Limited, 2020, 2017.

[29] Gibson LJ Ashby MF . Cellular solids: structure and properties. 2nd ed. Cambridge: Cambridge University Press Cambridge, 1997.

[30] McGrath MP Gao J Tang J , et al. Development of a residuum/socket interface simulator for lower limb prosthetics. Proc IMechE, Part H: J Engineering in Medicine 2017; 231: 235–242. 10.1177/0954411917690764 28164748

[31] Cagle JC Reinhall PG Allyn KJ , et al. A finite element model to assess transtibial prosthetic sockets with elastomeric liners. Med Biol Eng Comput 2018; 56: 1227–1240. 29235055 10.1007/s11517-017-1758-z PMC5999538

[32] Lee WC Zhang M Mak AF . Regional differences in pain threshold and tolerance of the transtibial residual limb: including the effects of age and interface material. Arch Phys Med Rehabil 2005; 86: 641–649. 15827912 10.1016/j.apmr.2004.08.005

[33] Steer JW Worsley PR Browne M , et al. Key considerations for finite element modelling of the residuum–prosthetic socket interface. Prosthet Orthot Int 2021; 45(2): 138–146. 33176573 10.1177/0309364620967781

[34] Roberts AP Garboczi EJ . Elastic properties of model random three-dimensional open-cell solids. J Mech Phys Solids 2002; 50: 33–55.

[35] Radcliffe CW . The biomechanics of below-knee prostheses in normal, level, bipedal walking. Artif Limbs 1962; 6: 16–24. 13972953

[36] Össur. Pressure pads. In: Össur (ed.) Össur liners and sockets [online]. Reykjavik, Iceland: Össur, 2021, p. 96.

[37] Sanders JE Greve JM Clinton C , et al. Changes in interface pressure and stump shape over time: preliminary results from a trans-tibial amputee subject. Prosthet Orthot Int 2000; 24: 163–168. 11061203 10.1080/03093640008726539

[38] Chen AI Balter ML Chen MI , et al. Multilayered tissue mimicking skin and vessel phantoms with tunable mechanical, optical, and acoustic properties. Med Phys 2016; 43: 3117–3131. 27277058 10.1118/1.4951729 PMC4884191

[39] Turner S Belsi A McGregor AH . Issues faced by people with amputation(s) during lower limb prosthetic rehabilitation: a thematic analysis. Prosthet Orthot Int 2022; 46: 61–67. 34789709 10.1097/PXR.0000000000000070 PMC8865619

